# Role of FGF2 in Promoting Osteogenic Differentiation for Craniofacial Bone Regeneration

**DOI:** 10.1007/s40883-025-00447-4

**Published:** 2025-06-25

**Authors:** Xianrui Yang, Peter X. Ma

**Affiliations:** 1 Department of Orthodontics, College of Dentistry, University of Florida, Gainesville, FL 32610 USA; 2 Schools of Dentistry, Engineering, and Medicine, University of Michigan, 1011 North University Avenue, Ann Arbor, MI 48109 USA

**Keywords:** Fibroblast growth factor II, Craniofacial bone, Mesenchymal stromal cells, Drug delivery, Tissue regeneration

## Abstract

**Abstract:**

Fibroblast growth factor II (FGF2), or basic fibroblast growth factor (bFGF), is an important regulator in bone and craniofacial development. FGF2 regulates cell survival, proliferation, migration, multilineage differentiation, and stemness in stromal cells. While there is broad interest in utilizing FGF2 for bone and craniofacial tissue repair and regeneration, the literature and reported data are often inconsistent or even controversial due to its multifunctional nature. Therefore, the outcomes are dependent on dose, duration, timing of administration, spatiotemporal pattern of the FGF2 delivery, and the microenvironment. This review paper aims to discuss FGF2 signaling and its related pathways, as well as mechanisms *in vitro*, *in vivo*, and in clinical applications of FGF2 in inducing osteogenic differentiation of human mesenchymal stromal cells (hMSCs) for craniofacial bone regeneration.

**Lay Summary:**

Fibroblast growth factor II (FGF2) regulates bone and craniofacial development, including cell survival, proliferation, migration, multilineage differentiation, and stemness in stromal cells. While there is broad interest in utilizing FGF2 for bone and craniofacial tissue repair and regeneration, the literature and reported data are often inconsistent or even controversial due to the multifunctional nature of FGF2. The outcomes depend on dose, duration, timing of administration, spatiotemporal pattern of the FGF2 delivery, and the microenvironment. This review paper aims to discuss FGF2 signaling pathways, their crosstalk, drug delivery vehicles, and scaffolds for craniofacial bone.

## Introduction

Human mesenchymal stromal cells (hMSCs) can differentiate into multiple cell types, including osteoblasts [1–7]. The process by which hMSCs develop into bone-forming cells, i.e., osteoblasts and osteocytes, is called osteogenic differentiation [8]. It is a fundamental mechanism underlying bone regeneration, but the process is governed by a complex interplay of transcription factors, signaling pathways, and molecular cues [2]. The osteogenic differentiation of hMSCs offers potential solutions for bone-related degenerative conditions and holds immense promise in bone regenerative medicine for millions of patients [9]. Thus, further understanding the molecular regulation of hMSCs in osteogenesis is crucial for future clinical applications.

Fibroblast growth factor II (FGF2) [10–20], also known as the basic fibroblast growth factor (bFGF) [21–33] is an important regulator for cell survival, proliferation, migration, multilineage differentiation, and stemness in stromal cells [25, 34]. FGF2 was initially identified for its mitogenic properties. Later on, its role as a growth factor was discovered, followed by its function to regulate cell behavior as a multifunctional mediator, which can be applied in tissue engineering [18]. The functions of FGF2 include promoting differentiation to mature osteoblasts and enhancing the proliferation of immature osteoblasts, which can help with bone formation and regeneration if applied appropriately [35]. Since the proliferation of immature osteoblasts may inhibit the subsequent differentiation, the dual roles of FGF2 for osteogenic differentiation and bone regeneration have been debated. It was reported that FGF2 promotes preosteoblast differentiation if bound to FGF Receptor-1 (FGFR1) or FGFR3 and promotes both preosteoblast and osteoblast differentiation if bound to FGFR2 [36]. On the other hand, FGF2 was found to inhibit mineralization if it binds to FGFR3 and inhibits osteoblast differentiation and mineralization if it binds to FGFR1 [36]. The intricate control of FGF2 over osteogenic commitment and craniofacial bone regeneration is affected by various factors, including delivery methods, cell types, and its multifaceted interactions with extracellular and intracellular signaling cascades [37].

Roles of FGF2 in osteogenic differentiation of hMSCs have been widely investigated, but ambiguities exist in the studies, and no clear mechanism has been determined. Some researchers suggest that FGF2 promotes osteogenic differentiation of hMSCs [38] while others suggest it inhibits the process [16]. Studies found decreased bone volume and bone mass in mice when FGF2 was knocked out. The MSCs from FGF2 knockout mice showed suppressed osteogenic differentiation and increased adipogenesis *in vitro* [20]. Other researchers found that FGF2 inhibits osteogenesis due to its potential to maintain cell stemness [35, 39]. In addition, many studies have tried to figure out a definitive mechanism of FGF2 regulation, but it has turned out to involve complicated interactions. FGF2 interacts with various intracellular pathways such as wingless-related integration site (WNT), mitogen-activated protein kinase (MAPK), and bone morphogenetic proteins (BMP) to modulate cellular responses and regulate osteogenesis [40].

Based on current knowledge, the osteogenic effect of FGF2 on hMSCs is still unclear because it depends on various conditions, including cell culture, interacted signaling, the mode of administration, dosage, duration, and spatial-temporal applications. For example, the effects and mechanisms are different if FGF2 is loaded with biomaterials located between the matrix and cells compared to the continuous injection of FGF2 into the culture medium [41]. This review delves into the various factors and signaling pathways related to the FGF2 function in regulating osteogenic differentiation of hMSCs in cell culture, as well as in bone regeneration in mouse models and clinical trials, shedding light on the implications for FGF2-based therapies in the clinic.

## Potential Biomaterials and Mechanisms for Controlling the Release and Delivery

The release and delivery of FGF2 in a spatiotemporally controlled manner are crucial for stimulating craniofacial bone regeneration. Researchers have tried various approaches, including hydrogels, polymeric scaffolds, microspheres, and nanoparticles, as well as surface modification of these biomaterials, to improve delivery [42].

**Fig. 1:**
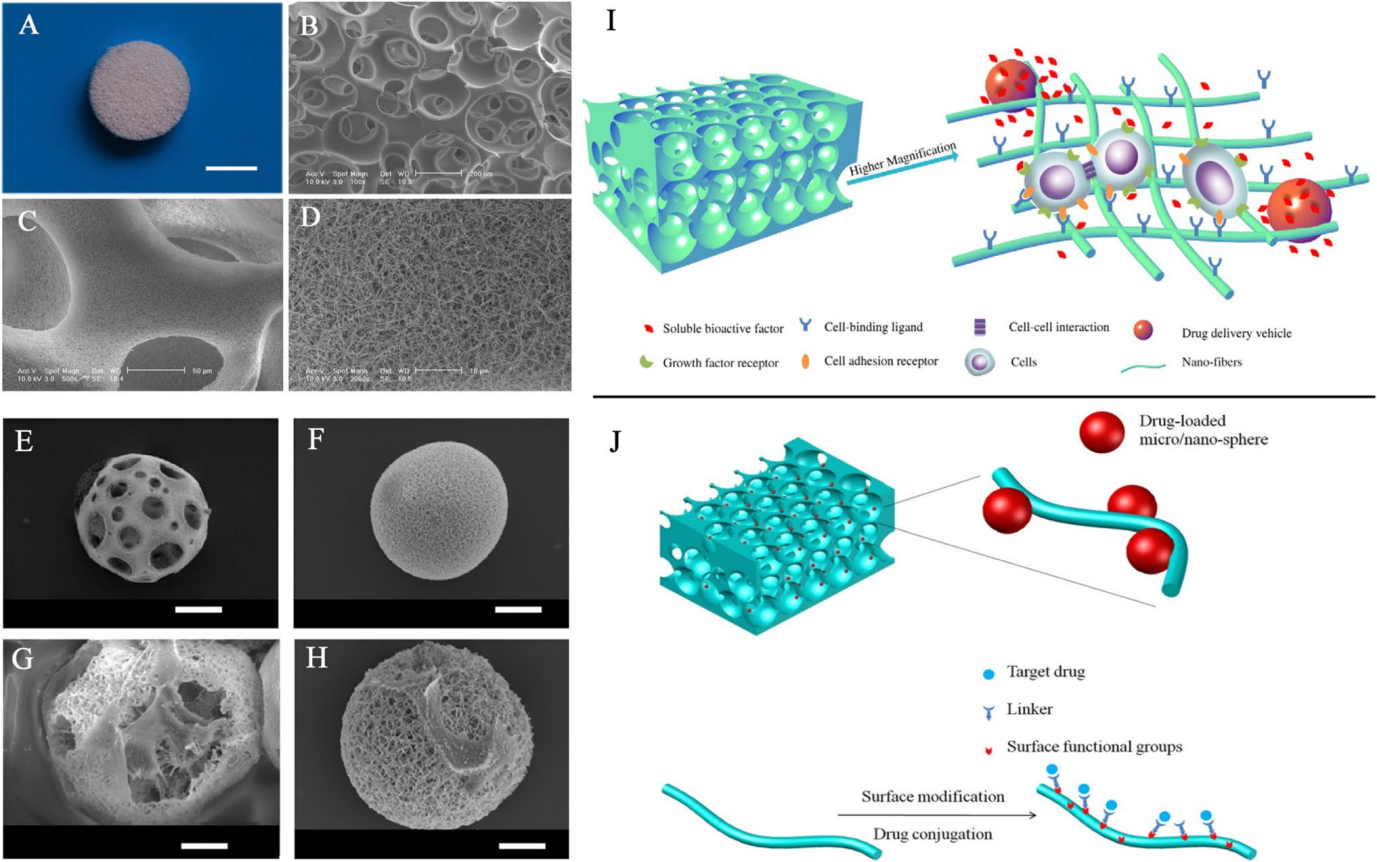
Structures of nanofibrous biomaterials and their modifications for drug delivery. Nanofibrous biomaterials hold significant promise as scaffolds, enabling the loading of stem cells and bioactive agents through surface pore immobilization and covalent conjugation, thereby enhancing drug delivery for bone regeneration. **A** NF-PLLA scaffold under macroscopic observation. **B** NF-PLLA scaffold at low magnification under SEM observation. **C**, **D** NF-PLLA scaffold at a higher magnification (from Wei et al. [44] with permission). **E** Structure of nanofibrous spongy microspheres (NF-SMS). **F** Structure of nanofibrous microspheres (NF-MS). **G** NF-SMS seeded with hDPSCs. **H** NF-MS seeded with hDPSCs (from Kuang et al. [43] with permission). **I** Schematic illustration of a biomimetic nanofibrous scaffold with the integrated osteogenic microenvironment, which can deliver bioactive agents and/or stem cells in a three-dimensionally controlled manner. **J** Schematic illustration showing the typical nanofiber-based drug delivery strategies through covalent conjugation of bioactive agents or pore surface immobilization of bioactive agent-loaded particles (from Zhang et al. [45] with permission)

Nanofibrous biomaterials, mimicking fibrous extracellular matrix proteins, have shown great potential as scaffolds for bone regeneration. The modification of nanofibrous poly (l-lactic acid) (NF-PLLA) scaffold, nanofibrous spongy microspheres (NF-SMS), and nanofibrous microspheres (NF-MS) makes them suitable for delivery applications. Researchers have loaded stem cells and bioactive agents into these biomimetic scaffolds through covalent conjugation or pore surface immobilization (Fig. 1) [43–45].

Biodegradable microspheres and nanoparticles have been incorporated into scaffolds or injected into tissue directly after encapsulating FGF2 for localized delivery and release kinetics control [46, 47]. Other biomaterial examples of the delivery systems include hydrogels (water-swollen polymer networks) to encapsulate and release FGF by adjusting the composition and crosslinking density. They include natural polymers such as hyaluronic acid, alginate, fibrin, and collagen and synthetic polymers such as poly(ethylene glycol) diacrylate (PEGDA) [48]. It is important to develop the controlled release system for FGF2, maintaining its bioactivity during the encapsulation and release, and ensuring its delivery to target tissues in optimal concentrations. In addition, the route of administration and intended duration of therapy should be considered for a specific clinical application.

## Effects of the FGF2 Administration Method and Dosage on the Osteogenic Differentiation of hMSC

As a multifunctional growth factor, FGF2 plays crucial and complicated roles in regulating cellular processes associated with osteogenic differentiation [35]. The optimal FGF2 dosage and duration would ideally balance between differentiation and proliferation of stromal cells to maximize bone regeneration. The administration methods of either directly adding it in culture media or delivering it through biomaterials also affect the osteogenic results [49]. All these factors influence the osteogenic differentiation of hMSCs and are indispensable for effective craniofacial bone regeneration strategies. As shown in Table 1, we have summarized studies published from 2001 to 2021 about FGF2 regulation of the osteogenic differentiation of hMSCs. It includes hMSC sources and density, FGF2 dosage and duration, culture and differentiation medium, as well as co-factors, biomaterials, and administration methods, all of which could influence the FGF2 effect on hMSC differentiation (Table 1).

**Table 1:** Summary of research on FGF2 regulation of osteogenic differentiation in human mesenchymal stem cells (hMSCs)

Study ID	hMSC source	hMSC density	hMSC culture medium	hMSC differentiation medium	FGF2 dosage (ng/ml)	FGF2 duration(day)	FGF2 administration methods	Adding co-factors	Add biomaterials
Tsutsumi 2001	Bio-Whittaker Inc.	1 or 5×10^3^/cm^2^	DMEM	β-Glycerophosphate, dexamethasone, ascorbic acid	N/A	28	N/A	None	No
Akita 2004	Bio-Whittaker Inc.	1×10^4^/four 35 mm^2^	DMEM-LG	β-Glycerophosphate, dexamethasone, ascorbic acid	2.5	4	Add into medium	rhBMP2	No
Sotiropoulou 2006	Adult donors	1,5, 10, 25, 50, 100, 200 ×10^3^ cells/cm^2^	DMEM, MEM	β-Glycerophosphate, dexamethasone, ascorbic acid	0.01, 0.1, 1, 5, 10, 20	21	Add into medium	None	No
Go 2008	Human donors	1×10^4^/well (12-well plates)	αMEM	β-Glycerophosphate, dexamethasone, ascorbic acid	10	14	Add into medium	NGF	No
Ahn 2009	Cambrex Bio Science	2×10^4^/cm^2^	αMEM	β-Glycerophosphate, dexamethasone, ascorbic acid	5	14	Add into medium	JNK inhibitor	No
Fierro 2011	Lonza	1×10^4^/cm^2^; transduct 5000/cm^2^	αMEM	β-Glycerophosphate, dexamethasone, ascorbic acid	N/A	21	Lentiviral vectors	None	No
Hudalla 2011	Cambrex	2000/cm^2^	αMEM	β-Glycerophosphate, dexamethasone, ascorbic acid	1, 5	1,2,3	Add into medium	Heparin	Yes
Zheng 2011	Bone marrow aspirates	2×10^4^/well (100 μl)	DMEM	β-Glycerophosphate, dexamethasone, ascorbic acid	N/A	1,7,14,28	plasmid transfection	None	Yes
Biver 2012	Healthy donors	1×10^4^/cm^2^	DMEM	β-Glycerophosphate, dexamethasone, ascorbic acid, VD3	10	3,7,10,14	Add into medium	MAPK inhibtors	No
Mostafa 2012	Patient donors	N/A	DMEM	GP, Dex, bFGF, BMP2	10	15,25	Add into medium	Dex,BMP2,Vit D3	No
Hagmann 2013	bone marrow of donors	5×10^5^/cm^2^	DMEM-LG	β-Glycerophosphate, dexamethasone, ascorbic acid	10	21	Add into medium	None	No
Lee 2014	ATCC	1×10^5^/ml	DMEM	N/A	100	1,4,7,14,21,28	Sequential delivery	BMP2	Yes
Moon 2015	Lonza Corporation	1×10^4^/well (24-well plates)	α-MEM	β-Glycerophosphate, dexamethasone, ascorbic acid	50, 100, 200 (ng)	1,2	Immobilized on BCP	None	Yes
Lee 2015	Gift from Dankook	5000/100 μl	Lonza/Commercial	N/A	0, 10, 20, 40, 60,100	2,4,6	DNA construct	None	None
Lei 2015	AllCells	6×10^3^/well (96-well plates)	DMEM	β-Glycerophosphate, dexamethasone, ascorbic acid	20	2	Sequential delivery	BMP2	Yes
Levy 2015	Healthy donors	5×10^3^/well (6-well plates)	DMEM	β-Glycerophosphate, dexamethasone, ascorbic acid	10, 50, 100	2	Free vs Conjugated	None	Yes
Moon 2015	Lonza Corporation	1×10^4^/well (24-well plates)	αMEM	β-Glycerol phosphate, ascorbic acid, dexamethasone	10, 50, 100, 200, 400 (ng)	1,2, 7, 14	Grafting onto BCP	None	Yes
Blache 2016	Healthy human donors	1.5×10^6^/mL	αMEM	HEPES, ascorbic acid, β-glycerol phosphate	50	14	Add into 3D culture	BMP2	Yes
Mishra 2016	RoosterBio	2.5×10^4^/cm^2^(PPF); 3.6×10^5^/scaffold	hMSC	Dexamethasone, b-glycerophosphate, ascorbic acid	5	1,3,7	Add into medium	PDGF-BB,EGF,BMP4,6,7	Yes
Lennon 2018	Healthy adult donors	4500/cm^2^	DMEM-LG	Ascorbic acid 2-phosphate, dexamethasone	1, 10	21	Add into medium	PDGF	None
Rajendran 2019	Lonza	1.6×10^4^/well	PromoCell/Commercial	PromoCell/Commercial	20	14	Soluble vs Immobilized	None	Yes
Suliman 2019	Healthy donors	Seed 3–5×10^3^/cm^2^ (12-well plates)	N/A	StemPro/Commercial	5, 10	21	Add into medium	None	Yes
Tae 2019	Catholic Institute	1×10^6^/microwells	αMEM	L‐Glutamine, ascorbic acid, dexamethasone	30, 60, 90	1,3,5,7,13	Treated with	None	Yes
Hao 2020	Cyagen Biosciences Inc	Seed 2×10^5^/6-well dishes	DMEM	Cyagen Biosciences/Commericial	5, 10, 15, 20, 25, 30	7	Add into medium	TNFa	No
Zhang 2020	Placental amniotic membrane	Seed 1×10^5^/ml	N/A	N/A	0, 10, 20, 40	14	Lentivirus transfected	Platelet-rich plasma	No
Lai 2021	Wharton Jelly	1×10^6^/ml (scaffold)	ScienCell/Commercial	Stem-Pro/Commericial	500	3,7,14	Releasing	None	Yes
Zhou 2021	Cyagen biosciences	Seed 1×10^5^/sample	αMEM	N/A	10 (ng)	1,4,7	Load into GelMA	BMP2	Yes

This table presents detailed information on studies investigating the effects of FGF2 on the osteogenic differentiation of human mesenchymal stem cells (hMSCs). Each entry includes the study ID (first author’s last name and publication year), the hMSC source, cell density, culture medium, differentiation medium, FGF2 dosage, treatment duration, and administration method. It also lists any co-factors and biomaterials used in each study. The studies span from 2001 to 2021, with hMSCs obtained from commercial sources or human donors. FGF2 was applied in varying dosages and durations, with or without co-factors and biomaterials, to stem cells cultured at different densities. Variations in cell culture and osteogenic differentiation media across studies may also contribute to differences in observed outcomes

### Dosage and duration

The dosage and duration of FGF2 treatment in cell culture are fundamental determinants of its effects. Most researchers believe lower concentrations promote cell proliferation and maintain undifferentiated stem cells, while higher concentrations stimulate osteogenic differentiation and mineralization [50]. Studies found that the effect of FGF2 is greater at the very early stage of bone regeneration and diminishes with a prolonged administration mode [51]. The timing of adding FGF2 can also affect its lineage commitment and subsequent osteogenic differentiation. If FGF2 is provided in the medium during the early period of osteogenic differentiation, it promotes stromal cell proliferation and expansion. It is advantageous to obtain a larger population of cells for subsequent differentiation, but excessive proliferation may negatively impact differentiation potential and retain cells in a more undifferentiated state. On the other hand, when FGF2 is provided in later stages, it promotes the maturation and mineralization of osteoblasts, leading to the formation of functional bone-forming cells [52]. Thus, the intricacies of FGF2 dosage and timing responses underscore the importance of precision control of dosing and duration to induce the osteogenic differentiation of hMSCs and craniofacial bone regeneration.

One group of researchers harvested human mesenchyme-derived progenitor cells from young and old patients and cultured them with different FGF2 concentrations of 0.0016, 0.016, 0.16, and 1.6 ng/ml for 4, 24, 28, and 72 h. They found that there was more proliferation in human cells at lower concentrations (0.0016 ng/ml) of FGF2 than in mouse cells but less proliferation from older bone cells [53]. A second group of researchers used 10, 20, and 40 ng/ml FGF2 for hMSC culture and found that a lower concentration of FGF2 showed a positive effect on differentiation [54]. A third group used 5, 10, 15, 20, 25, and 30 ng/ml FGF2 and found that FGF2 had no obvious function in enhancing osteogenic gene expression, but it could ameliorate impaired osteogenesis [13]. A fourth group used 10, 20, 40, 60, and 100 ng/ml FGF2 and found the mRNA expression of bone markers was upregulated when cells were treated with FGF2 for 6 days before osteogenic induction, which indicates pretreatment with FGF2 during culture increases osteogenic potential [23]. A fifth group did the experiments using 0.01, 0.1, 1, 5, 10, and 20 ng/ml bFGF and believed low concentration bFGF promoted the proliferation rate of hMSCs and increased bFGF concentration promoted mineralization [28]. The varying findings of these experiments have resulted from different experimental conditions and environments [55].

### Administration method

The most common administration methods include continuous supplementation of the cell culture medium and delivery using biomaterials. Adding FGF2 directly into the culture medium over prolonged periods can affect cellular behavior, including proliferation and differentiation rates [35]. Nevertheless, the delivery method that involves biomaterials such as scaffolds or carriers can control FGF2 release to mimic the natural spatial-temporal biochemical environment within bone, guiding osteogenic differentiation and bone regeneration *in vivo* [56].

The stability of FGF2 is also affected by its administration method. Combined with biomimetic scaffolds for the delivery of FGF2, they stabilize and maximize FGF2’s biological activity during a certain period by countering the inherent instability of the FGF2 protein in aqueous solutions [57]. Porous polymer scaffolds, hydrogels, or biodegradable microspheres were found to enhance the osteogenic activity of FGF2 [30] because they can protect FGF2 from proteolysis or inactivation and prolong its biological half-life *in vitro* and *in vivo* [21, 55, 58, 59].

## FGF2-FGFR Signaling Regulation and Its Interaction with Other Signaling Pathways

There are four FGF receptors (FGFRs), FGFR1, FGFR2, FGFR3, and FGFR4, which can bind FGFs and then combine with heparan sulfate proteoglycans (HSPG) [32] or heparin to activate FGFs-FGFR signaling pathways to induce cellular responses [60]. Among them, FGFR2 has two isoforms, FGFR2b and FGFR2c. FGFR2b contains exon 8 and binds to FGF1, 3, 7, 10, and 22, exclusively expressed in epithelial cells, while FGFR2c contains exon 9 and binds to 1, 2, 4, 6, 9, 17, and 18, exclusively expressed in mesenchymal cells [52]. FGF2 has various isoforms that are highly expressed in bone tissues. The FGF2 isoforms of high molecular weight (22, 23, and 24 kD) are localized in the nucleus. They act as transcriptional factors and upregulate associated genes of impaired mineralization, such as FGF-23 and SOST [61, 62]. The FGF2 isoform of low molecular weight is located in the cytoplasmic region and is membrane-associated. It promotes osteoblast differentiation and mineralization through related signaling, such as BMP and WNT pathways [14]. FGF2 binds equally to FGFR1 and FGFR2 and then activates the downstream signal transduction differentially, which may lead to the progenitor proliferation and osteogenic differentiation of stem cells [63]. Some cues and signals can trigger the binding of FGF2 to FGFRs and their interactions with other signaling, such as mechanical signals, specific enzymes, growth factors, and inflammatory cytokines around the bone defect areas [64, 65].

**Fig. 2:**
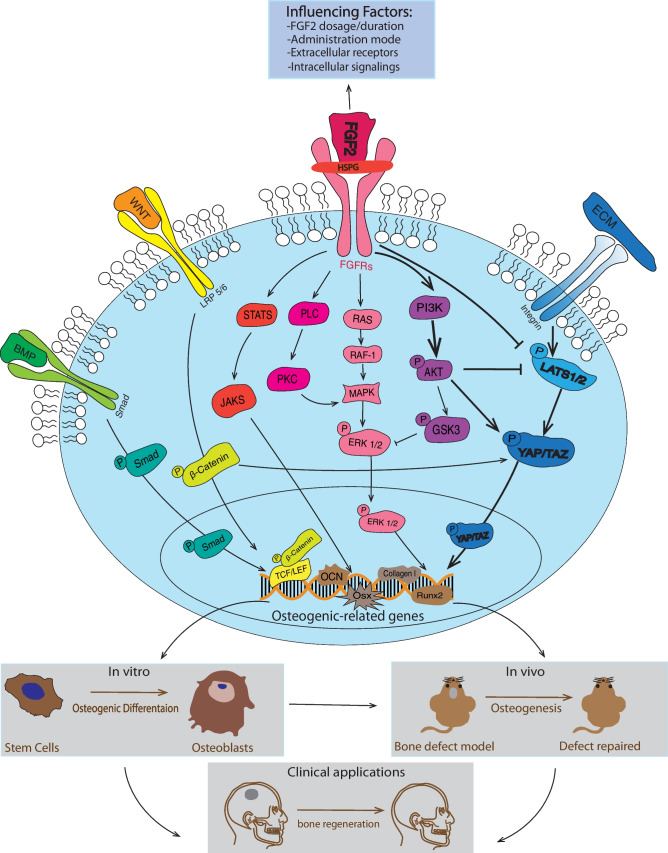
FGF2/FGFRs pathways and their interacting signaling in regulating osteogenic differentiation and craniofacial bone regeneration. FGF2/FGFRs regulate osteogenic-related genes through multiple signaling pathways and are affected by factors such as FGF2 dosage, duration, and administration mode. The binding of HSPG (heparan sulfate proteoglycan) and FGF2 on the extracellular domain causes FGFR dimerization to activate and autophosphorylate tyrosine residues of the cytoplasmic domain, which leads to intracellular pathways. The main involved FGF2/FGFR signaling pathways are PI3K-AKT-GSK3 (phosphatidylinositol 3 kinase-protein kinase B-glycogen synthase kinase 3), PLCγ-PKC (phospholipase C to phospholipase Cγ-protein kinase C), RAS-MAPK-ERK1/2 (rat sarcoma-mitogen-activated protein kinase-extracellular signal-regulated kinase 1 and 2), and STATs-JAKs (signal transducer and activator of transcription proteins-Janus kinase). They also interact with other signaling, such as WNT/β-catenin and BMP/Smad pathways, to activate the expression of osteogenic-related genes. FGF2/FGFRs can inhibit LATS1/2 to affect YAP/TAZ activation, which is also regulated through ECM/integrin and PI3K-AKT signaling. The activated genes will induce the *in vitro* osteogenic differentiation of stem cells, *in vivo* osteogenesis of mice craniofacial bone defects, and clinical applications for patients who need craniofacial bone regeneration

The binding of FGF2 to FGFRs through a heparan sulfate glycosaminoglycan binding site limits the FGF2 diffusion through the extracellular matrix (ECM) [66]. Heparin increases FGF’s affinity, while high FGF levels activate FGFR without heparan [67]. The binding of HSPG (heparan sulfate proteoglycan) and FGF2 on the extracellular domain causes receptor dimerization to activate and autophosphorylate tyrosine residues of the cytoplasmic domain, which leads to lots of signal pathways that regulate cell proliferation, differentiation, migration, survival, and shape [11]. Four major intracellular signaling pathways are activated upon binding of FGFs to FGFRs: PI3 K-AKT-GSK3 (phosphatidylinositol 3 kinase-protein kinase B-glycogen synthase kinase 3) [62], PLCγ-PKC (phospholipase C to Phospholipase Cγ-protein kinase C), RAS-MAPK-ERK1/2 (rat sarcoma-mitogen-activated protein kinase-extracellular signal-regulated kinase 1 and 2), and STATs-JAKs (signal transducer and activator of transcription proteins-Janus kinase) [26, 32, 68]. The ultimate function of these signaling pathways also depends on the integration and crosstalk with other signaling pathways (Fig. 2).

### Regulation of FGF2/FGFR-PI3K-AKT-GSK3 Signaling

The PI3K-AKT-GSK3 signaling pathway is activated by FGF2/FGFR binding and autophosphorylation of the tyrosine residues with FGFR’s cytoplasmic domain, which plays crucial roles in regulating various cellular processes such as cell growth, survival, proliferation, differentiation, and metabolism [69]. The activated FGFR recruits and activates PI3K and then phosphorylates the lipid PIP2 (phosphatidylinositol 4,5-bisphosphate) to generate PIP3 (phosphatidylinositol 3,4,5-trisphosphate) within the cell membrane [70]. The PIP3 is a critical secondary messenger that recruits proteins with the pleckstrin homology domain, such as AKT, to the cell membrane [70]. After the AKT is activated, it can phosphorylate various downstream substrates in a multitude of cellular processes. For example, GSK3 is one key target of AKT, which is inhibited by phosphorylated AKT activation. The inactivated GSK3 showed significant implications for cellular activities such as regulating gene expression, cell survival, and cell cycle progression [71]. The FGF2/FGFR-PI3K-AKT-GSK3 is important in transducing signaling from ECM to the nucleus to regulate cellular responses, dysregulation of which may cause certain diseases such as cancer and developmental disorders [72].

### Regulation of FGF2/FGFR-PLCγ-PKC Signaling

Activated FGFR phosphorylates and activates phosphoinositide phospholipase C (PLCγ), and then PLCγ acts on membrane phospholipids to generate secondary messengers IP3 (inositol-trisphosphate) and DAG (diacylglycerol). IP3 triggers calcium ion release from intracellular stores to increase cytoplasmic calcium levels [73]. The elevated cytoplasmic calcium, in conjunction with DAG, activates the serine/threonine kinase protein kinase C (PKC). Then, PKC translocates to the cell membrane, where it becomes activated and phosphorylates various target proteins [74]. The activation of PKC and its downstream targets mediates a range of cellular responses, including gene expression, cell survival, proliferation, migration, and differentiation [52]. The mechanism of premature cranial suture ossification in mice and humans caused by FGFR2 overexpression is the activation of PLCγ and PKC to increase osteoblast gene expression in osteoblast precursor cells [75].

### Regulation of FGF2/FGFR-RAS-MAPK-ERK1/2 Signaling

FGF2/FGFR can also activate the growth factor receptor-bound protein 2 (Grb2) [68] and subsequently activate the GTPase RAS protein [76]. The RAS protein switches between an active GTP-bound state and an inactive GDP-bound state. The activation of RAS also leads to its downstream effector RAF1 (rapidly accelerated fibrosarcoma 1) protein activation [77]. The RAF1 phosphorylates and activates MAPKK (mitogen-activated protein kinase kinase), which is also known as MEK [78]. MAPKK then phosphorylates and activates the members of the MAPK (mitogen-activated protein kinase) family, such as ERK1/2 (extracellular signal-regulated kinase 1/2). The activated ERK1/2 translocates to the nucleus to phosphorylate various target proteins to induce cellular responses for activating osteogenic-related genes [79].

### Regulation of FGF2/FGFR-STATs-JAKs Signaling

The FGF2/FGFRs-STATs-JAKs pathway is related to the modulation of cell proliferation, differentiation, inflammation, and immune responses [80]. STATs are transcription factors that play roles in mediating gene expression in response to extracellular signals. JAKs are kinases that associate with cytokine receptors and are essential for transmitting signals from the cell membrane to the nucleus [81]. The activated FGFR recruits and activates JAKs, and then JAKs phosphorylate STATs, leading them to form dimers and translocate to the nucleus [82]. The STAT dimers bind to specific DNA sequences, which are known as response elements, within the promoters of target genes in the nucleus. The binding changes gene expression, enabling cells to carry out specific functions based on the extracellular signals [81].

### FGF2/FGFR Signaling Interacts with the WNT/β-Catenin Signaling

FGF2 affects the WNT/β-catenin pathway by upregulating and stabilizing β-catenin, a key transcriptional coactivator in the canonical WNT pathways [61]. The elevated WNT signaling results in β-catenin accumulation in the cytoplasm, preventing its degradation and allowing it to translocate to the nucleus. Then, β-catenin binds to the TCF/LEF family (transcription factors of T-cell factor/lymphoid enhancer-binding factor), which activates target genes associated with osteogenic differentiation, such as osteocalcin and collagen type I [83]. On the other hand, the WNT/β-catenin signaling can also stimulate the expression of FGF receptors and FGF ligands to induce some cellular responses involving tissue development and homeostasis [31]. The interplay between FGF2/FGFRs and WNT/β-catenin pathways synergistically regulates bone formation and skeletal development [84]. Studies found knockout FGF2 in osteoblast culture to reduce bone nodule formation, and then the bone formation can be rescued through WNT/β-catenin signaling by adding exogenous FGF2 [12].

### FGF2/FGFR Signaling Interacts with the BMP/Smad Signaling

The FGF2/FGFRs and BMP/Smad signaling pathways complement each other in promoting osteogenesis [85]. FGF2 stimulates the expression of BMP ligands, particularly BMP2 and BMP4, as well as their receptors. The binding of BMP ligands to their receptors activates Smad proteins, which translocate into the nucleus to act as transcription factors that regulate osteogenic gene expression [86]. Studies found that high doses of FGF2 do not increase BMP receptor expression, while low doses of FGF2 strengthen bone formation by increasing Smad 1 expression, a major downstream effector in the BMP signaling pathway [8]. Moreover, researchers found that the treatment with FGF2 alone suppressed osteogenic differentiation, while osteogenic differentiation was achieved when FGF2 was combined with BMP2 treatment [87]. The delivery of FGF2 and BMP2 showed stronger osteogenesis in culture than those without any growth factors or with a single administration of FGF2 or BMP2 [23]. The release of FGF2 and BMP2 in a sequential manner was efficient for osteogenic differentiation, while reversing the release sequence of FGF2 and BMP2 reduced osteogenesis [4].

### FGF2/FGFRs Interact with the YAP/TAZ (Yes-Associated Protein and Transcriptional Coactivator with PDZ-Binding Motif)

Together with YAP [88–91], TAZ [89, 91–94] is the effector protein of the Hippo pathway, which is crucial in sensing mechanical cues from the microenvironment and regulating cell proliferation and mesenchymal stromal cell differentiation [95]. TAZ can activate myoblast and osteoblast differentiation and inhibit adipocyte differentiation by interacting with Runx2 (Runt-related transcription factor 2) to stimulate the Runx2-mediated gene transcription and interacting with PPARγ (peroxisome proliferator-activated receptor gamma) to inhibit PPARγ-mediated gene transcription [94, 96]. The activation of Runx2-mediated gene transcription is essential for osteoblast differentiation and to promote bone matrix production [97]. Studies found that FGF2 signaling regulates osteogenesis by increasing TAZ mRNA expression, which ERK activation stimulates. Depletion of TAZ blocks FGF2-mediated osteogenesis, and inhibition of ERK blocks TAZ expression [98].

On the other hand, FGF2/FGFRs signaling also activates AKT, which phosphorylates and sequesters YAP/TAZ in the cytoplasm. This phosphorylation prevents their nuclear translocation, limiting transcriptional activities [89]. Activating YAP/TAZ promotes cell proliferation, while their cytoplasmic retention by FGF2-induced AKT activation favors osteogenic differentiation [99]. The interaction between the FGF2/FGFR pathway and the YAP/TAZ pathway creates a balance between cell proliferation and differentiation. This interplay not only influences stem cell fate decisions but also provides a strategy to optimize bone tissue regeneration by leveraging the regulatory network of these pathways [100].

### FGF2/FGFR Signaling Interacts with the LATS1/2 (Large Tumor Suppressors 1/2) Signaling

The LATS1/2 signaling pathway is one of the components in the Hippo signaling pathway and functions in cell proliferation and apoptosis to regulate tissue regeneration, organ size, and tumorigenesis [101]. The serine/threonine kinases LATS1 and LATS2 are key components of the Hippo pathway. When the pathway is activated, LATS kinase phosphorylates and inhibits the transcriptional co-activators YAP and TAZ to cause the sequestration of YAP/TAZ in the cytoplasm to prevent their translocation to the nucleus and interaction with transcription factors involved in cell survival and proliferation [92]. FGF2 can affect the Hippo pathway by influencing the expression of LATS1/2 kinase and YAP/TAZ mediated gene expression [78]. FGF2 activates downstream effectors such as AKT, which phosphorylate and inhibit LATS kinases, thereby preventing the phosphorylation of YAP/TAZ [91]. This inhibition of LATS1/2 caused by FGF2 helps to release YAP/TAZ to translocate to the nucleus and enhance their transcription coactivator function to promote the expression of genes that are essential for bone matrix formation and mineralization [92]. The interplay between FGF2 and LATS1/2 signaling pathways creates a permissive environment for osteogenic differentiation, and inhibition of LATS1/2 by FGF2 ensures that YAP/TAZ are available to activate osteogenic gene expression. Understanding the interaction between FGF2/FGFR and LATS1/2 pathways provides insights into strategies for optimizing bone tissue engineering approaches [90]. By manipulating the activities of LATS1/2 kinases and influencing the localization and function of YAP/TAZ, the FGF2/FGFRs pathway contributes to the orchestration of cellular responses that promote osteogenesis [91].

## FGF2 Regulation of hMSC Osteogenic Differentiation In Vitro

The administration of FGF2 *in vitro* shows inconsistent effects on osteogenic differentiation of hMSCs, depending on FGF2 concentration, administration methods, exposure duration, and other manipulation strategies. Studies found that moderate concentrations of FGF2 enhance osteogenic differentiation, while excessive amounts lead to undesired results of other lineages’ differentiations [35]. Thus, it is essential to determine the balance of optimal concentration of FGF2 to induce desired osteogenic differentiation of hMSCs.

Exposing hMSCs to FGF2 during the early stages of osteogenic differentiation is usually beneficial, followed by reduced exposure or withdrawal to encourage cells to transition toward osteoblast differentiation [102]. FGF2 induces bone formation, targeting younger cells but not mature cells [35, 103]. Also, FGF2 increases cell growth in the early stage, which is an important step to expand the cell population for subsequent osteogenic differentiation and bone regeneration. FGF2 maintains bone marrow stromal cells in an immature state to sustain their osteogenic differentiation by inducing less alkaline phosphatase [5, 7, 14, 17, 20, 27, 38, 104–110].

The controlled delivery approach has been used to optimize FGF2 function in osteogenic differentiation of stromal cells. FGF2 is initially provided to promote hMSC proliferation and commitment to the osteogenic lineage, then the FGF2 is removed from the culture medium to encourage osteoblast differentiation, allowing cells to progress along the osteogenic pathway [7]. Certain biomaterials or scaffolds can control the release of FGF2 and mimic the natural three-dimensional tissue microenvironment, which may be beneficial for orchestrating osteogenic differentiation [111].

Researchers found that FGF2 promotes hMSC osteogenic potential since it enhances cell proliferation and life span, but the effects vary with hMSC concentrations. It is believed that FGF2 has greater effects on the low seeding density of hMSC culture (1000 cells/cm^2^) compared to high-density hMSC culture (5000 cells/cm^2^) [112]. If cells were passaged from the high initial cell density, MSC from the 3^rd^, 6^th^, and 9^th^ passages had similar levels of alkaline phosphatase and calcium with or without FGF2, irrespective of the passage numbers. If hMSCs were passaged from the lower initial cell density, there was a higher calcium content with FGF2 than without FGF2. The calcium content and the mRNA expression of osteopontin, osteocalcin, and bone sialoprotein were higher [112].

**Fig. 3:**
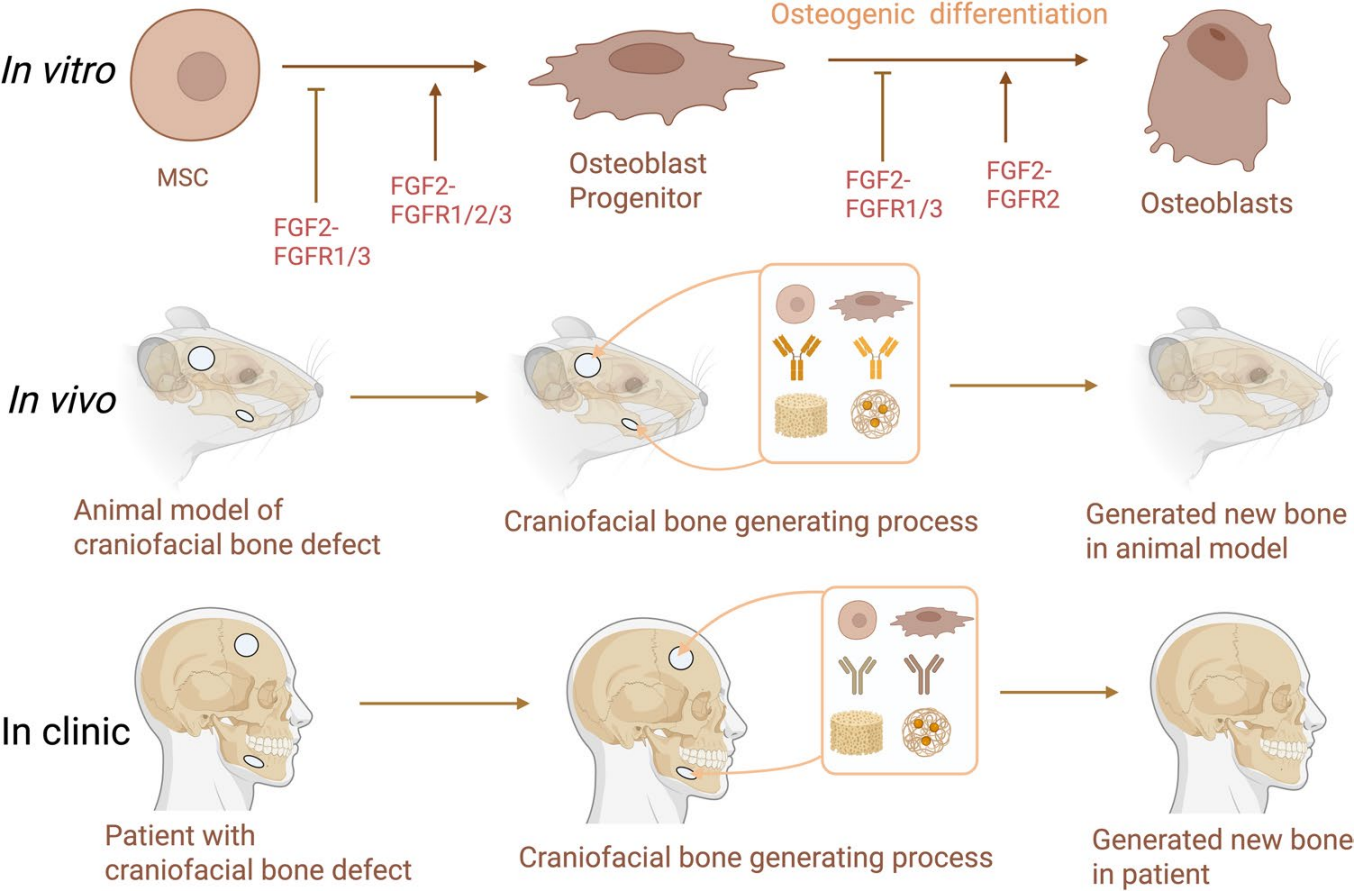
Effects of FGF2 on MSC differentiation in vitro and craniofacial bone regeneration in vivo and in clinical settings. FGF2 binding to different FGFRs can either promote or inhibit various stages of MSC differentiation in vitro. In vivo studies in mice, as well as clinical applications in patients with craniofacial bone defects, have shown that combining FGF2 with MSCs and biomaterials can effectively support craniofacial bone regeneration

On the other side, some researchers reported that FGF2 down-regulated mineralization in various stem cells. This phenomenon was linked to FGF2’s role in maintaining stemness and inhibiting differentiation, particularly toward the osteogenic lineage [113, 114]. By interacting with its receptors, FGF2 activates intracellular pathways that promote self-renewal and inhibit differentiation. It also suppresses the expression of genes involved in mineralization and bone matrix formation, which are crucial for MSCs to differentiate into mature osteoblasts [115]. Therefore, FGF2’s effect on mineralization underscores the delicate balance between cell proliferation and differentiation. While FGF2 sustains stemness and proliferation, it interferes with the transition to osteogenic differentiation, where cells need to exit the cell cycle, express osteogenic genes, and promote mineralization [116] (Fig. 3).

## Role of FGF2 *In Vivo* for Bone Regeneration in Mouse Models

The spatiotemporal patterns of FGF2 function *in vivo* vary from those under culture conditions. One reason is that FGF2 signaling *in vivo* is affected by interacting with many other cytokines and signaling pathways [117]. Another reason is that the administration modes in mouse models affect the reactivity in inducing osteogenesis in the bone defect areas [118]. Also, the different bone defect configurations *in vivo* require different FGF2 functions and efficiencies [119]. Furthermore, different dosages and durations were used by different groups in their studies based on various aims, which made it hard to reach a consensus [13, 33, 109].

Researchers discovered that FGF2 played dual roles in bone formation in transgenic mouse models. FGF2 knockout mice showed reduced bone formation and skeletal dwarfism, which displayed a loss of connecting rods and reduced trabecular plate-like structures [120]. The knockout mice also showed reduced bone mass, and their bone marrow mesenchymal cells showed poor osteogenic potential compared to control mice [17]. This phenotype happened due to the deficiency of FGF2, leading to reduced osteogenesis and increased adipogenesis [20]. However, FGF2 overexpression in mice also led to skeletal defects such as flattening and shortening bones due to decreased osteoblast differentiation [19].

Some studies found that the administration of FGF2 *in vivo* promoted cranial regeneration and shortened the craniofacial bone repair time by influencing cell recruitment, proliferation, angiogenesis, and osteogenic differentiation [30, 121, 122]. FGF2 led to superior bone formation and increased bone defect filling in aged mice, suggesting that FGF2 can be used to boost bone regeneration in defect areas with impaired angiogenic potential or limited numbers of native osteoprogenitor cells [123]. Some researchers believe that FGF2 recruits progenitor cells, including mesenchymal stromal cells and osteoprogenitor cells, to the site of injury or defect in mouse models and then stimulates osteogenic differentiation of these cells by working synergistically with other growth factors and signaling pathways [124]. Other researchers believe that FGF2 promotes the growth of blood vessels that supply nutrients and oxygen to regenerate bone tissue, as well as facilitate the deposition and mineralization of extracellular matrix components such as proteoglycans and collagen, which are essential for bone matrix formation and development [125].

However, FGF2 has a short half-life of 12 h *in vivo* due to its instability, i.e., being degraded by proteolytic enzymes after being thawed [57]. Thus, researchers fabricated multi-layered PLLA nanosheets loaded with rhFGF2 to allow sustained release for two weeks to improve bone regeneration after implantation in murine-critical bone defects [118]. Some researchers fabricated FGF2-loaded gelatin hydrogel scaffolds for bone tissue regeneration because they can mimic the way that FGF2 is stored in the ECM. They stated that the vascularization process was unchanged when FGF2 solution was injected directly into mouse bone defect areas, while neovascularization was enhanced when FGF2 was incorporated into a gelatin hydrogel. In addition, the slower resorption rate of hydrogels with less water concentration of 77.5% was more efficacious in maintaining longer-term FGF2 release compared to a hydrogel with a high water concentration of 95.9% [126, 127]. The biomaterials in the FGF2 delivery systems made it feasible and controllable for manipulating *in vivo* osteogenesis (Fig. 3).

## Combination of FGF2 and Other Factors for Craniofacial Bone Regeneration

As reviewed thus far, FGF2 has multiple potentials in facilitating bone regeneration, such as enhancing proliferation, promoting angiogenesis, recruiting MSCs, and enhancing their osteogenic differentiation. However, these functions are dependent on FGF2 dose, application timing, duration, and *in vitro* and *in vivo* environments. Its osteogenic function is especially sensitive to the dose, timing, and duration. Therefore, its use with a more robust osteogenic factor and an ECM-mimicking scaffold is of particular attraction. Rambhia et al. used controlled-release poly(lactic-co-glycolic-acid) (PLGA) nanospheres to distinctly tailor BMP7 and FGF2 doses and temporal release profiles in a collagen geometry-mimicking nanofibrous scaffold [128]. Their results showed that BMP7-induced bone formation was accelerated by a relatively higher FGF2 dose (100 ng/scaffold) delivered at a faster release rate or by a relatively lower FGF2 dose (10 ng/scaffold) at a slower release rate in an *in vivo* bone regeneration model. In contrast, a very high dose of FGF2 (1000 ng/scaffold) inhibited bone regeneration under all conditions. *In vitro* and *in vivo* data suggest that FGF2 improved BMP7-induced bone regeneration by coordinating FGF2 dosage and release kinetics to enhance stem/progenitor cell migration, proliferation, and angiogenesis, resulting in optimal cranial bone regeneration. Similarly, other growth factors that promote angiogenesis and mesenchymal stromal cell proliferation, such as platelet-derived growth factor (PDGF) [129] and vascular endothelial growth factor (VEGF) [130], may also be considered in combination with various BMPs to achieve more robust bone regeneration.

## Clinical Applications of FGF2 for Treating Craniofacial Bone Defects in Patients

FGF2 is promising for clinical applications in treating patients with craniofacial bone defects because it stimulates osteogenesis and angiogenesis to enhance bone defect repair. Studies show that FGF2 expression is reduced in older people, resulting in decreased intrinsic proliferation potential of hMSCs and increased diseases [15]. hMSC can be obtained from human bone marrow repeatedly, so transplantation of hMSCs can provide a way to treat related diseases, including arthritis, intrinsic muscular dystrophies, osteoporosis, cardiac diseases, degenerative nerve diseases, and periodontal diseases [131]. With the development of biomaterials, it becomes possible to modulate the dosage, duration, and stability of FGF2 in defect areas [118]. However, there are no standard methods to deliver FGF2, nor are there optimal dosages and durations for FGF2 applications for clinical bone regeneration.

Before widespread clinical applications, rigorous preclinical studies and clinical trials are necessary to assess the safety, efficacy, and long-term outcomes of FGF2-based treatments. Patient selection and appropriate case assessment are crucial since not all craniofacial defects may benefit from FGF2-based therapies, and treatment plans should be customized to individual patient needs [132]. It is also essential to consider ethical issues, patient consent, and transparent communication about the potential benefits and risks of FGF2-based treatments [35]. The osteogenic and angiogenic properties of FGF2 make it a promising candidate for treating craniofacial bone defects in patients, but thorough research, clinical trials, and personalized treatment plans are necessary to ensure safe and effective outcomes. Collaborations between researchers, clinicians, and regulatory authorities are required to advance FGF2-based therapies for clinical craniofacial bone regeneration.

rhFGF2 has recently been used in clinical treatment for craniofacial bone regeneration [18]. There are at least 17 randomized controlled trials evaluating the clinical efficacy of rhFGF2 in treating periodontal defects [133]. For example, a double-blind randomized controlled trial included 253 patients treated with 0.2%, 0.3%, and 0.4% rhFGF2, or placebo for their periodontal intra-bony defect regeneration surgery [134]. Those who received FGF2 treatments showed significantly more bone regeneration radiographically compared with the placebo, of which 0.3% concentration demonstrated the best performance with no adverse effects [134]. In addition, a randomized controlled trial that included 30 patients also demonstrated significant improvement in pocket depth reduction and clinical attachment level after treatment with FGF2 compared to their control sites [135]. Another randomized controlled trial with various FGF2 concentrations together with β-tricalcium phosphate scaffolds showed that patients treated with 0.3% or 0.4% rhFGF2 resulted in 71% success for bone regeneration and clinical attachment gain compared to 45% success with the 0.1% FGF2 group and controls [136]. Two systematic reviews and meta-analyses have been published to discuss the efficacy and efficiency of rhFGF2 treatment [133, 137]. One evaluated the effect of rhFGF2 and rhPDGF-BB for treating deep intra-bony periodontal defects [137]. The study included FGF2 concentrations of 0.03%, 0.1%, 0.2%, 0.3%, and 0.4%, and showed that FGF2 benefits clinical bone fill [137]. The other one summarized six randomized controlled trials with the same concentrations of 0.03%, 0.1%, 0.2%, 0.3%, and 0.4% and demonstrated that the clinical outcomes of rhFGF2 treatments were dose-dependent. The higher the FGF2 concentration, the better the bone regeneration [133]. The above-discussed roles of FGF2 are illustrated in Fig. 3. Several recent clinical trials have been carried out to test FGF2-based therapies. For example, Imamura et al. carried out a 12-month randomized controlled trial for periodontal regenerative therapy comparing rhFGF2 alone with rhFGF2 in combination with carbonate apatite granules. They found no differences between the two groups [138]. Kojima et al. conducted a randomized clinical trial using rhFGF-2 alone and rhFGF-2 combined with autogenous bone for periodontal regeneration. They concluded that rhFGF2 promotes hard tissue regeneration in intraosseous defects in both groups [139]. Seshima et al. did a 4-year extended follow-up of a randomized controlled trial comparing periodontal regenerative therapy using rhFGF2 alone versus rhFGF2 with deproteinized bovine bone mineral. They found favorable clinical, radiographic outcomes, and patient-reported outcomes in both groups maintained for at least four years [140]. They did not find differences between the groups.

Although many clinical studies have been conducted and shown positive outcomes of FGF2 treatment for bone regeneration, there remain challenges and limitations in translating FGF2-based therapies from preclinical models to clinical applications, such as dosage control, delivery methods, and long-term safety [141–143]. The optimal dosage is critical for FGF2 treatment, as both underdosing and overdosing lead to suboptimal results or adverse effects [141]. The delivery method is also a concern, as the protein’s short half-life necessitates advanced formulation strategies to ensure localized and sustained activity [142]. Long-term safety is another concern when applying FGF2 treatment widely. While short-term studies demonstrate favorable results, the long-term application of FGF2 raises concerns over potential pro-oncogenic effects and immune responses [143]. More investigations and trials are required to address these safety considerations. In sum, while FGF2-based treatments hold substantial promise for bone regeneration in the clinics, a deeper understanding of their pharmacodynamics, targeted delivery, and long-term safety is essential for successful clinical translation.

## Discussion and Perspectives

Research shows that FGF2 has unique roles in craniofacial bone regeneration, as well as in the development of craniofacial bone [144]. During the craniofacial bone regeneration process, FGF2 facilitates the mobilization of MSCs and periosteal progenitors to the bone defect sites, enhances their differentiation into osteoblasts to promote bone formation, and stimulates new blood vessel formation, which is essential for the delivery of oxygen and nutrients to regenerate bone tissue [142]. Although studies found that FGF2 can promote the expansion of osteoprogenitor cells and early osteoblast differentiation, the optimal concentration and duration are critical [145]. Otherwise, negative effects of FGF2 in osteogenesis are also reported in the literature [146]. In addition, FGF2 was identified to modulate cranial suture patency, and its dysregulation was linked to craniosynostosis; it was also found to promote the formation of the palatal shelves and prevent the occurrence of cleft palate during craniofacial development [147, 148].

The dual roles of FGF2 in hMSC behavior, promoting stemness and inhibiting mineralization and enhancing osteogenic differentiation, have implications for clinical applications in craniofacial bone tissue regeneration. The detailed mechanism underlying FGF2’s dual roles is still not fully explored, but a few hypotheses have been proposed. Different signaling pathways may be activated at different stages with certain dosages of FGF2 to promote hMSC stemness or enhance osteogenic differentiation [149]. Maintaining the multipotent state of hMSCs by FGF2 can occur through the activation of MAPK-ERK1/2 to promote pluripotency-associated gene expression, such as SOX2, OCT4, and NANOG, or through the inhibition of RUNX2 expression [150]. On the other hand, FGF2 can enhance the BMP and WNT pathways, which stabilize RUNX2 in late-stage differentiation, leading to increased ALP activity and matrix mineralization [151]. Another potential explanation is that FGF2 can bind different FGFR isoforms to alter downstream signaling; for example, FGFR1 favors stemness via ERK-dominance while FGFR2/3 activation tips the balance toward osteogenesis via PI3K/AKT and STAT signaling [152]. Finding strategies to tailor the competing roles of FGF2 will facilitate clinical applications of FGF2 therapies.

Current studies often show contradictory results in terms of FGF2 function and the mechanism, but each of them may be affected by possible biases or may be accurate only under certain specific circumstances. The absence of consensus is probably due to some key factors that have yet to be addressed for a clearer understanding of FGF2’s dual role. One of the factors could be differences in experimental conditions. Some studies have used high-dose FGF2 treatments for extended periods, which may induce a proliferative effect, preventing cells from differentiating into osteoblasts [153]. Conversely, short-term or low-dose FGF2 exposure has been associated with priming hMSCs for differentiation under osteogenic stimuli [154]. Another factor could be the variability in cell sources. hMSCs obtained from different tissues may display unique differentiation potentials and responses to growth factors, likely linked to variations in receptor expression profiles and inherent differentiation abilities [155]. However, how cell lineage and tissue origin influence the effectiveness of FGF2 as a therapeutic agent, particularly in the context of tissue engineering of craniofacial bone, is still not clear and needs more exploration [142]. In addition, variability in delivery methods can also potentially affect the outcomes of FGF2-based therapies. Some studies used soluble FGF2 in a culture medium; some used biomaterial-based delivery systems such as hydrogels or scaffolds to regulate FGF2 activity in a more controlled and localized manner [156, 157]. However, variations in delivery kinetics, scaffold composition, and FGF2 release profiles can lead to differing biological responses. Research showed that the burst release of FGF2 enhances cell proliferation at the cost of differentiation, while sustained release might promote a more balanced outcome between stemness and osteogenic differentiation [158–161]. Therefore, in summary, there are no universally accepted conclusions on the spatiotemporal effects of FGF2 treatments. While FGF2 holds significant promise for stem cell-based regenerative therapies, particularly in the context of craniofacial bone regeneration, the conflicting results in research indicate a more nuanced understanding of the dual roles is necessary.

The effects of FGF2 depend on the dosage, durations of exposure, mode of administration, stage of cell differentiation, and *in vitro* or *in vivo* models. Overall, lower concentrations of FGF2 promote cell proliferation but not hMSC differentiation. In contrast, higher FGF2 concentrations within a certain range stimulate osteogenic differentiation and mineralization. Too high concentrations inhibit osteogenesis. Moreover, adding FGF2 early in cell culture can promote stem cell proliferation and expansion. The expansion benefits subsequent differentiated function for regeneration owing to a larger cell population, but excessive expansion possibly negatively impacts differentiation potential and maintains a more undifferentiation state of MSC. In contrast, adding FGF2 at a later stage promotes the maturation and mineralization of osteoblasts *in vitro*, which leads to the formation of functional bone-forming cells. However, long-term treatment or high dosages of FGF2 inhibit bone regeneration. Short-term or low-dose treatment enhances bone regeneration, which can also be explained by the proliferation function of FGF2, which is essential at the beginning stage.

The mechanisms of the positive or negative effects of FGF2 on the osteogenic differentiation of MSC and bone regeneration need to be further studied, where more defined parameters, including dosage, duration, administration methods, and interactions of signaling pathways, should be considered. Overexpressed FGF2/FGFR2 signaling activates PLCγ and PKC pathways to increase osteoblast gene expression. It also activates ERK1/2 to translocate to the nucleus to phosphorylate various target proteins for activating osteogenic-related genes. Moreover, FGF2 stimulates WNT ligands and receptors to activate the WNT/β-catenin pathway to enhance osteogenic differentiation. It also stimulates BMP ligands and receptors to upregulate the BMP/Smad pathway to strengthen bone formation. Besides, FGF2 activates ERK to increase TAZ mRNA expression to regulate osteogenesis. The FGF2/FGFRs signaling also activates AKT to phosphorylate YAP/TAZ in the cytoplasm and prevent their nuclear translocation, which favors osteogenic differentiation. Phosphorylation of AKT inhibits LATS1/2 kinases, thereby possibly preventing the phosphorylation of YAP/TAZ in the nucleus and interacting with transcription factors involved in cell proliferation to activate osteogenic gene expression. The detailed and unknown biological mechanisms of these signaling pathways’ interactions and functions need to be further investigated.

The way in which FGF2 is delivered may have a non-negligible impact on its effect as it decides its stability and temporal pattern. FGF2 alone or combined with scaffold and other molecules shows varying results. Identifying the optimal delivery methods and dosage within a given time for craniofacial bone regeneration is the key challenge to the success of FGF2-regulated bone tissue engineering therapy. Incorporating FGF2 into bone grafts or scaffolds has shown positive results in promoting craniofacial bone regeneration [162]. The controlled release of FGF2 from grafts or scaffolds enhances osteogenic differentiation and bone matrix deposition at the defect site, as well as enhances vascularization in the defect region, supporting nutrient and oxygen delivery to regenerating bone tissues in humans [163]. Controlled delivery of FGF2, mimicking the natural healing process, can optimize its effects and reduce the risk of excessive FGF2 concentrations to avoid unwanted effects [10].

## Conclusions

We reviewed the mechanisms of FGF2 regulation of human mesenchymal stromal cells and their osteogenic differentiation and summarized the potential biomaterials, biological signaling, and related factors for osteogenic differentiation *in vitro*, *in vivo*, and in the clinic. Many factors, such as FGF2 dosage, mode of administration, duration of exposure, and the stage of cell differentiation, affect the function of FGF2. Lower doses may enhance cell proliferation, while higher doses may promote differentiation and mineralization, but an appropriate proliferation of cells benefits subsequent differentiation and bone regeneration. Nevertheless, a very high dose for a long-term treatment inhibits osteogenesis. While there has been significant knowledge developed during the past few decades, there is no universal consensus on the spatiotemporal effects of FGF2. There are also potential biases among scientists of different backgrounds. Some of the findings may only stand true under specific circumstances. More studies are needed to further elucidate the roles and mechanisms that FGF2 plays in the osteogenic differentiation of stromal cells, which may pave the way to the success of utilizing FGF2 and stromal cells in treating patients who suffer from craniofacial bone loss or diseases in the future. Scaffolds and controlled release systems will likely play key roles in regulating the stromal cells and enabling the desired spatiotemporal delivery of FGF2 in such therapies. Future research should further investigate the mechanisms of various biomaterials used to deliver FGF2 in a controlled manner, to identify the most effective delivery methods and determine the optimal dosage of rhFGF2. Long-term safety of FGF2 therapy should also be evaluated. In addition, studies should carefully control experimental conditions, assess the impact of different cell sources, and explore innovative delivery systems. Clarifying the mechanistic interactions between FGF2 and other signaling pathways is crucial to understanding its dual roles and developing more effective clinical strategies for craniofacial bone tissue regeneration.

## Data Availability

All data generated or analyzed during the current study are available from the corresponding author upon reasonable request.

## Abbreviations

*FGF2*: Fibroblast growth factor II
*bFGF*: Basic fibroblast growth factor
*WNT*: Wingless-related integration site
*MAPK*: Mitogen-activated protein kinase
*BMP*: Bone morphogenetic proteins
*hMSC*: Human mesenchymal stem/stromal cells
*BCP*: Bathocuproine
*PVA*: Polyvinyl alcohol
*PLGA*: Poly(lactic-co-glycolic-acid)
*PCL*: Polycaprolactone
*FGFR*: Fibroblast growth factor receptor
*HSPG*: Heparin sulfate proteoglycans
*ECM*: Extracellular matrix
*PI3 K-AKT-GSK3*: Phosphatidylinositol 3 kinase-protein kinase B-glycogen synthase kinase 3
*PLCγ-PKC*: Phospholipase C to Phospholipase Cγ-protein kinase C
*RAS-MAPK-ERK1/2*: Rat sarcoma-mitogen-activated protein kinase-extracellular signal-regulated kinase 1 and 2
*STATs-JAKs*: Signal transducer and activator of transcription proteins-Janus kinase
*IP3*: Trisphosphate
*DAG*: Diacylglycerol
*PKC*: Protein kinase C
*Grb2*: Growth factor receptor-bound protein 2
*GTP*: Guanosine triphosphate
*GPTases*: GTP-binding proteins
*RAS*: Rat sarcoma
*RAF1*: Rapidly accelerated fibrosarcoma 1
*MAPKK/MEK*: Mitogen-activated protein kinase kinase
*ERK1/2*: Extracellular signal-regulated kinase 1/2
*TCF/LEF*: Transcription factors of T-cell factor/lymphoid enhancer-binding factor
*YAP*: Yes-associated protein
*TAZ*: Transcriptional coactivator with PDZ-binding motif
*Runx2*: Runt-related transcription factor 2
*PPARγ*: Peroxisome proliferator-activated receptor gamma
*LATS1/2*: Large tumor suppressors1/2
*PDGF*: Platelet-derived growth factor
